# High-resolution spectroscopy of [H,C,N]^+^: II. Ground state rotational spectrum of HCN^+^ (*X̃*^2^Π)

**DOI:** 10.1039/d5cp04204f

**Published:** 2025-12-03

**Authors:** Weslley G. D. P. Silva, Philipp C. Schmid, Divita Gupta, Sven Thorwirth, János Sarka, Oskar Asvany, Stephan Schlemmer

**Affiliations:** a I. Physikalisches Institut, Universität zu Köln Zülpicher Str. 77 50937 Köln Germany silvaw@ph1.uni-koeln.de schlemmer@ph1.uni-koeln.de

## Abstract

The rotational spectrum of HCN^+^ has been measured between 270 and 520 GHz in a cryogenic ion trap instrument employing a double resonance vibrational-rotational scheme based on leak-out spectroscopy. A total of four transitions with quantum numbers ranging from *J*″ = 2.5 to 5.5 have been observed for the lowest-energy spin–orbit state ^2^Π_3/2_ of this open-shell cation, which has an inverted ^2^Π electronic ground state. The transitions show well-resolved *Λ*-doubling components each displaying complex hyperfine splittings due to the presence of two nuclei with non-zero spins, ^1^H (*I* = 1/2) and ^14^N (*I* = 1). The accurate transition frequencies and ground state spectroscopic parameters determined for HCN^+^ in this work will enable radio astronomical searches of this important cation in the interstellar medium.

## Abstract 2

The ground-state rotational spectrum of the elusive open-shell radical cation HCN^+^ has been measured for the first time using a vibrational-rotational double-resonance approach based on leak-out spectroscopy.

## Graphical abstract

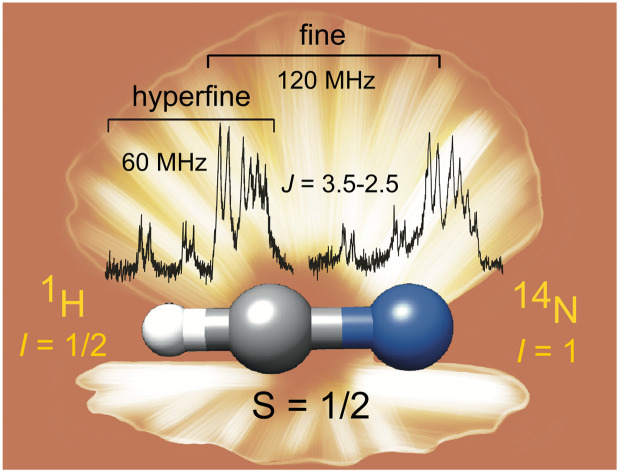

## 1. Introduction

Molecules containing a cyano group (CN) are crucial components of both terrestrial and interstellar chemistry.^1^ Together with CH, the CN radical was one of the first molecules detected in the interstellar medium (ISM) by radio astronomy.^2^ Given its fundamental importance for chemistry and astronomy, the CN radical, as well as its ionic variants CN^−^ and CN^+^, have become the subjects of several laboratory spectroscopic and astronomical studies. Because molecular ions are typically highly reactive and hard to produce in large quantities in the laboratory, the pure rotational spectra of CN^−^ ^3^ and CN^+^ ^4^ were only reported more than 30 years after the first rotational laboratory work on neutral CN.^5^ Based on the millimeter-wave spectroscopic work of Gottlieb *et al.*,^3^ transitions corresponding to CN^−^ were identified in the radio spectra collected toward the envelope of the carbon star IRC+10216.^6^ Unfortunately, no astronomical detection of CN^+^ has been reported to date.

The next fundamental members of the cyanide family, neutral hydrogen cyanide (HCN) and its higher energy isomer hydrogen isocyanide (HNC), are well-known species both in the laboratory and space (see *e.g.*, the Cologne Database for Molecular Spectroscopy, CDMS,^7^ and McGuire^1^ and references therein). However, their primary cations HCN^+^ and the lower energy isomer HNC^+^ have been elusive to astronomers to date, even though they are considered by astrochemical models key intermediate species driving nitrogen chemistry in the ISM.^8^ One example of their role as important intermediates is the gas-phase reaction of HCN^+^/HNC^+^ with H_2_ that leads to the formation of HCNH^+^ + H, as recently re-measured at low temperatures in the laboratory.^9^ HCNH^+^ is also a well-known interstellar molecule.^10–14^ To date, high-resolution spectroscopic data on the HCN^+^ and HNC^+^ intermediates, in particular from rotational spectroscopic investigations which usually support astronomical detections, have not been reported in the literature.

Aiming to overcome the lack of spectroscopic data on HCN^+^ and HNC^+^, in the first paper of this series of high-resolution spectroscopic works (paper I), we report the observation of rotationally resolved infrared bands for both HCN^+^ and HNC^+^. The measured bands include the fundamental CH stretching vibration *ν*_1_ and the lower energy Renner-Teller component (Σ) of the *ν*_1_ + *ν*_2_ combination band of HCN^+^, as well as the fundamental NH stretching vibration of HNC^+^.^15^ This thorough investigation allowed accurate spectroscopic constants for several vibrational states of both ions to be determined for the first time.

Building on paper I,^15^ in this second work, we present the first pure rotational spectrum measured for a [H,C,N]^+^ species, the HCN^+^ cation. The rotational transitions were recorded in a 22-pole cryogenic ion trap apparatus employing a double-resonance vibrational-rotational scheme^16^ based on leak-out spectroscopy.^17^ Owing to the open-shell nature of HCN^+^ and to the presence of two atoms with non-zero nuclear spins (^1^H and ^14^N), the observed transitions exhibit complex patterns from resolved fine and hyperfine splittings, as shown in detail below.

## 2. Methods

### 2.1. Experimental section

The experiments reported here were conducted using the 22-pole cryogenic ion trap instrument, COLTRAP, which has been described in detail previously.^18^ The HCN^+^ ions were generated in a storage ion source *via* electron impact ionization (*E*_e^−^_ = 30 eV) of stable methyl cyanide (CH_3_CN). The produced *m*/*z* = 27 ions were then extracted from the source, mass selected using a quadrupole mass filter, and trapped inside the 4 K 22-pole trap.^19^ Upon entering the trap, the HCN^+^ ions were stopped and thermalized to the cryogenic temperature by collisions with He buffer gas (*n* ≈ 10^13^ cm^−3^) which was added to the trap continuously. Additionally, Ne was pulsed into the trap in a 1 : 3 Ne : He mixture using a piezoelectrically actuated valve at the beginning of each trapping cycle to serve as the collision partners for the leak-out process of the HCN^+^ ions. Once trapped, the spectra of HCN^+^ were recorded using leak-out spectroscopy, as described in detail previously.^17,20^

The COLTRAP experiments started by revisiting the rovibrational spectrum of the *ν*_1_ fundamental CH stretching band of HCN^+^. Although this band has been recently measured at a trap temperature of 35 K as discussed in detail in paper I,^15^ the colder trap temperature of COLTRAP typically leads to narrower spectral lines and may allow splittings to be better resolved (*e.g., Λ*-doubling). Furthermore, the accurate wavenumber positions of the rovibrational lines observed with the COLTRAP instrument are needed to support the millimeter-wave measurements. After remeasuring the band, the observed rovibrational lines were used in a double-resonance vibrational-rotational scheme which was employed to search for the pure rotational transitions of HCN^+^.

The double-resonance vibrational-rotational scheme based on LOS has been recently described in detail for HC_3_O^+^,^16^ and has also been successfully applied to other astrophysically relevant cations.^14,16,21–24^ During the double-resonance measurements, the wavenumber of the infrared beam was kept fixed on resonance with a rovibrational transition of the *ν*_1_ band (*e.g. ν*_1_ = 1, *J* + 1 ← *ν* = 0, *J*) generating a constant LOS signal. Then, additionally, millimeter-wave radiation was used to search for a pure rotational transition in the ground vibrational state (*ν* = 0, *J* ← *ν* = 0, *J* − 1) involving the common rotational quantum state (*ν* = 0, *J*) probed by the infrared laser. Upon hitting the rotational resonance, this procedure results in an increase in the population of the (*ν* = 0, *J*) level and thus, an enhancement in the LOS signal is observed. It is worth mentioning that other double-resonance level schemes are possible, in which a decrease in the LOS signal is observed by, for example, de-populating the (*ν* = 0, *J*) level. The rotational lines were recorded in individual measurements in which the millimeter-wave frequency was tuned in a given frequency window in fixed steps while monitoring the LOS signal. As the hyperfine structure of HCN^+^ is extended, relatively large step sizes of 20–100 kHz were used. The larger steps are typically used for initial searches, while the smaller ones are employed in the final measurements. The measurement counts were normalized using a millimeter-wave frequency-switching procedure, *i.e.* by dividing the number of HCN^+^ ions in the scanned frequency window by those at an off-resonance position. Thus, the resulting baseline is close to unity. The millimeter-wave power was lowered to sufficiently low levels to minimize power broadening effects. Examples of measured transitions are provided and discussed in detail below in the “Rotational measurements” section.

The infrared radiation was supplied by a continuous-wave optical parametric oscillator (cw-OPO, Toptica, model TOPO), whose frequency was measured continuously by a wavemeter/spectrum analyzer (Bristol Instruments, model 771A-MIR), which has a manufacturer-stated accuracy of ± 0.0006 cm^−1^. The measured laser power was on the order of a few hundred mW. The millimeter-wave radiation was generated using a Rb-clock-referenced microwave synthesizer (Rohde & Schwarz SMB 100A) driving an amplifier-multiplier chain (Virginia Diode Inc., VDI). With the setups available in Cologne, frequencies up to 1.1 THz can be reached.

### 2.2. Quantum chemical calculations

The experimental work described here was complemented by high-level quantum chemical calculations, performed using the CFOUR^25^ program package. Although a few theoretical works have been previously reported for HCN^+^,^26,27^ information on several molecular parameters relevant to deciphering the ground state rotational spectrum is still missing. The equilibrium structure of HCN^+^ was determined at the CCSD(T)^28,29^ level of theory using ROHF reference functions in the frozen core approximation with the aug-cc-pV5Z basis set.^30,31^ From the obtained equilibrium structure, the rotational (*B*_e_), centrifugal distortion (*D*_e_) as well as hyperfine constants for the non-zero spin nuclei ^1^H and quadrupolar ^14^N were obtained. The calculated hyperfine parameters include the Fermi contact (*b*_F_) and the diagonal dipole–dipole interaction tensor constants (*T*_aa_, *T*_bb_ and *T*_cc_) for both nuclei as well as the nuclear electric coupling quadrupole constants for the ^14^N nucleus (*eQq*). From the calculated *b*_F_, *T*_aa_, *T*_bb_ and *T*_cc_ constants, the Frosch and Foley hyperfine interaction constants *a* (nuclear spin–orbit), *b* (nuclear spin–electron spin), *c* (nuclear spin–electron spin dipole–dipole) and *d* (nuclear spin–electron spin dipole–dipole off diagonal term) can be derived for each nucleus using equations previously described in the literature.^32,33^ Similar computational approaches have been used to disentangle the hyperfine structure in the radio spectra observed for the larger siblings of HCN^+^, *i.e.*, HC_3_N^+^,^34^ HC_5_N^+^, and HC_7_N^+^.^35^ A summary of the computational results is provided below in Table 1. The optimized geometric parameters for the equilibrium structure of HCN^+^ are *r*_CN_ = 1.2159 Å and *r*_HC_ = 1.0914 Å.

## 3. Results and discussion

### 3.1. Rovibrational measurements of the fundamental *ν*_1_ C–H stretching vibration at 4 K

HCN^+^ is an open-shell molecular cation with a *X̃*^2^Π electronic ground state. The coupling of the electron spin (*S* = 1/2) and orbital (*Λ* = 1) angular momenta leads to two spin–orbit fine structure components; the lower-energy ^2^Π_3/2_ and the higher-energy ^2^Π_1/2_ state, which are separated by about 50 cm^−1^.^15,27^ In addition, the end-over-end rotation of the linear ion lifts the degeneracy of the orbital angular momentum (*Λ* = 1) resulting in further splitting of the rotational states, which is known as *Λ*-doubling. In paper I of this series,^15^ transitions from both spin–orbit states were observed in the rovibrational spectra of the fundamental *ν*_1_ CH stretching vibration of HCN^+^ measured at a trap temperature of 35 K. Furthermore, lines with higher *J* rotational quantum numbers (*J* = *N* + *S*) within each spin–orbit ladder showed well-resolved *Λ*-doubling components. Based on these observations, accurate spectroscopic parameters for the ground and *ν*_1_ vibrational states of HCN^+^ could be determined for the first time.

**Fig. 1:**
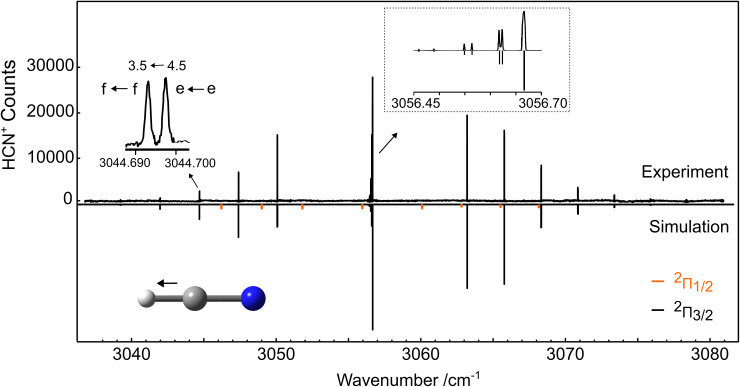
Comparison between the experimental (top) and simulated (bottom) spectra of the fundamental *ν*_1_ C–H stretching band of HCN^+^. The simulation was obtained using the spectroscopic constants derived in paper I of this series^15^ at a rotational temperature of 15 K. In the simulation, the lines from the lower-energy (^2^Π_3/2_) and higher-energy (^2^Π_1/2_) spin–orbit states are depicted in black and orange, respectively, but lines from the latter were not observed due to the low temperature conditions. The insets show the *J*′(*v*_1_) ← *J*″(*v*_0_) = 3.5 ← 4.5 transition in the P-branch, and the Q-branch progression in detail.

Guided by these first measurements,^15^ the *ν*_1_ band of HCN^+^ was readily identified in our experiments performed at a nominal trap temperature of 4 K. Owing to the low temperature conditions, only levels from the lower-energy spin–orbit state ^2^Π_3/2_ are populated. An overview of the 4 K spectrum together with a simulation of its spectral features obtained at a rotational temperature of 15 K using the PGOPHER program^36^ is provided in Fig. 1. In the simulation, lines from the higher-energy ^2^Π_1/2_ state are depicted in orange for illustration, but these are not observed in the cold spectrum as mentioned above. The zoomed-in regions in Fig. 1 show a portion of the Q-branch between 3056.45 and 3056.70 cm^−1^, and the P-branch *J*′(*v*_1_) ← *J*″(*v*_0_) = 3.5 ← 4.5 transition within the ^2^Π_3/2_ state in detail to highlight the well-resolved *Λ*-doubling components (labeled here also as *e* and *f*). *Λ*-doubling was observed for lines with quantum numbers *J* ≥ 2.5 in the Q-branch, and for all transitions in the P- and R-branches, with the smallest splittings being on the order of 0.002 cm^−1^. Overall, the observed lines in our spectrum show narrow Doppler widths; *e.g.*, the components of the P-branch *J*′(*v*_1_) ← *J*″(*v*_0_) = 3.5 ← 4.5 transition given in the inset of Fig. 1 have a full width at half maximum (FWHM) of about 60 MHz, which corresponds to a kinetic temperature of the ions of approximately 15 K. This temperature is higher than the nominal trap temperature of 4 K due to known heating effects.^37^

### 3.2. Rotational measurements

**Fig. 2:**
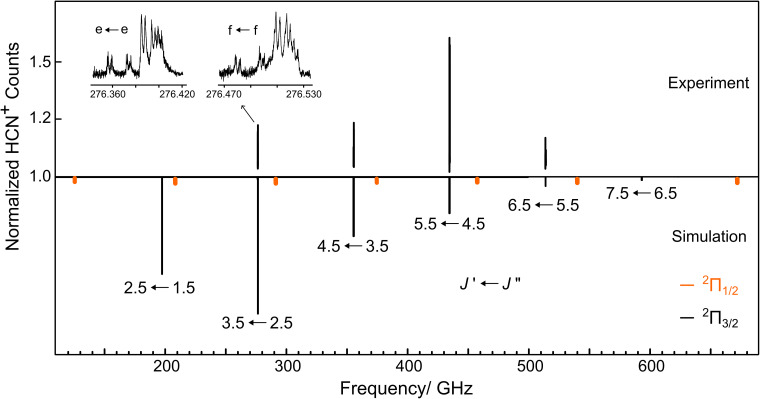
Overview of the pure rotational spectrum of HCN^+^ in the 180–700 GHz region. The observed transitions (top) are compared to a simulation (bottom) obtained at a rotational temperature of 15 K using the spectroscopic parameters from Table 1. In the simulation, lines from both ^2^Π_3/2_ (in black) and ^2^Π_1/2_ (in orange, not to scale) spin–orbit states are shown, but those belonging to the latter were not observed due to the low temperature conditions. The inset shows the *J*′ ← *J*″ = 3.5 ← 2.5 transition in detail with its resolvable *Λ*-doubling splitting, labeled here as *e* and *f*.

Based on the ground state spectroscopic parameters derived from the infrared measurements,^15^ we searched for pure rotational lines of HCN^+^ in the 270–520 GHz range. As HCN^+^ has a sizeable predicted dipole moment (3.63 D,^26^ 3.53 D this work at the CCSD(T)/aug-cc-pV5Z level), intense spectral features corresponding to different rotational transitions were readily observed in the vicinity of the initial predictions. In total, four rotational transitions (*J*″ = 2.5–5.5) with well-resolved *Λ*-doubling components were measured for the ^2^Π_3/2_ state. An overview of the experimental and simulated rotational spectra of HCN^+^ is given in Fig. 2. In the inset of Fig. 2, a zoom into the *J*′ ← *J*″ = 3.5 ← 2.5 transition is given to show its two *Λ*-doubling components (*e* and *f*) in the ground state, which are separated by about 120 MHz. Furthermore, it is evident in the zoomed-in spectra of Fig. 2 that each peak shows additional underlying splittings, spanning a window of roughly 60 MHz, which are thus not resolved in the infrared spectrum. These features can be attributed to magnetic hyperfine effects arising from the interaction between the non-zero spins of the ^1^H (*I* = 1/2) and quadrupolar ^14^N (*I* = 1) nuclei with the electron orbital and electron spin angular momenta. These interactions are known to lead to characteristic extended hyperfine structures, and were previously observed in the radio spectra collected toward TMC-1 for transitions attributed to the related HC_3_N^+^,^34^ HC_5_N^+^ and HC_7_N^+^ ^35^ open-shell cations, which also have ^2^Π electronic ground states.

**Fig. 3:**
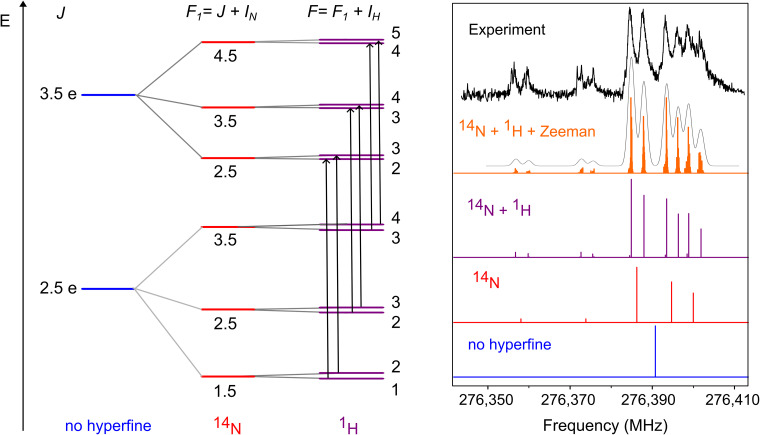
Portion of the energy level diagram of HCN^+^ (not to scale, left) in the ground vibrational state showing the hyperfine splitting of the *J* = 2.5 and *J* = 3.5 rotational states (*e*-component, ^2^Π_3/2_) due to both ^14^N and ^1^H nuclei. Selected allowed transitions with Δ*J* = 1, Δ*F*_1_ = 1, Δ*F* = 1 selection rules are illustrated in the diagram with black arrows. The transitions following these selection rules are the most intense features observed in the spectrum and they appear as six strong lines, as can be seen in the experimental spectrum shown on the right-hand side. A comparison between the simulated and observed ground state rotational spectrum for the *J*′ − *J*″ = 3.5*e* − 2.5*e* transition (infrared wavenumber fixed on resonance with the rovibrational *J* − *J*′ = 4.5*e* − 3.5*e* transition at 3068.324 cm^−1^) is also provided to illustrate the origin of the splittings observed. Convolving the Zeeman spectrum (orange trace) with Gaussian line shapes corresponding to a kinetic temperature of about 15 K (grey countour) reproduces best the observed spectrum.

To exemplify the complex hyperfine structure observed in the ground state rotational spectrum of HCN^+^ further, the *e*-component of the *J*′ ← *J*″ = 3.5 ← 2.5 transition is shown in detail in Fig. 3. Below the experimental spectrum, we provide different simulations to illustrate in a stepwise fashion the effect of each non-zero spin nucleus on the rotational structure of HCN^+^ (right-hand side). A correlated energy level diagram is also given on the left-hand side of Fig. 3. As shown in the diagram, each rotational level with a distinct quantum number *J* splits into three non-degenerate sub-levels due to interactions with the ^14^N nucleus. These sub-levels are further split into two additional levels when hyperfine contributions from the ^1^H nucleus are also taken into account, leading to a total of six non-degenerate rotational levels. To describe each nucleus, we used the following coupling scheme; *F*_1_ = *J* + *I*_N_ and *F* = *F*_1_ + *I*_H_ with *I*_N_ = 1 and *I*_H_ = 1/2. The selection rules for the R-branch ground state transitions measured are *e* ← *e* or *f* ← *f* with Δ*J* = 1; Δ*F*_1_ = 0, 1 and Δ*F* = 0, ±1. The most intense transitions are those with Δ*J* = 1, Δ*F*_1_ = 1 and Δ*F* = 1, which appear in the spectrum as six intense lines. For the *J*′ ← *J*″ = 3.5 ← 2.5 transition in Fig. 3, these features are well-resolved and can be readily seen in the spectrum between 276.38 and 276.41 GHz. The selection rules for the six strong transitions are highlighted in the energy level diagram using black arrows, and their expected spectral patterns are displayed in the purple trace of the simulated spectrum for reference. For transitions with quantum numbers *J*″ > 3.5, the hyperfine splitting becomes smaller and some of the components start to overlap (see an overview spectrum showing the hyperfine structure of all measured transitions in the SI). The less intense cluster of lines, appearing on the left-hand side of the main peaks (roughly 12–30 MHz below), match predictions for four hyperfine components which follow the Δ*J* = 1, Δ*F*_1_ = 0 and Δ*F* = 0 selection rules (purple trace). However, it is evident in the experimental spectrum that these lines, between 276.35 and 276.38 GHz, exhibit additional small splittings that are not described by our spectroscopic model. Although there could be four additional transitions, with selection rules Δ*F*_1_ = 0 and Δ*F* = 0, −1, in this range, they are predicted to be extremely weak and unlikely to appear in our low-temperature spectrum (see SI for more details).

In addition to fine and magnetic hyperfine effects, we speculate that the HCN^+^ transitions may also be influenced by the Zeeman effect from external magnetic fields, which could explain both the observed extra small splittings and the unusually broad, asymmetric line shapes. This reasoning is based on recent (unpublished) studies of other open-shell cations in our laboratory, including CCH^+^ (*X̃*^3^Π), OH^+^ (*X̃*^3^Σ^−^) and HCCD^+^ (*X̃*^2^Π), in which Zeeman splittings were observed in their ground state rotational spectra, and a magnetic field of about three times the Earth's magnetic field was estimated (1.8 × 10^−4^ T) from the experimental line shapes. The origin of this external magnetic field requires further detailed investigation but it could potentially arise from the magnetically levitated turbo pumps or from other magnetic parts in our experimental setup. By incorporating the Zeeman effect (*B*_field_ = 1.8 × 10^−4^ T) in our final spectroscopic model, a better reproduction of the observed spectrum can be obtained (see orange trace in Fig. 3).

### 3.3. Spectroscopic fits

The transition frequencies of the observed lines were determined by using a Gaussian line profile. As our line shapes are heavily influenced by the Zeeman effect splitting and some non-resolved hyperfine components, we assumed conservative uncertainties of 120 kHz for the determined line positions. In total, 71 individual hyperfine components (neglecting Zeeman) were assigned to 66 unique transition frequencies in the ground state rotational spectrum. For the less intense hyperfine components following Δ*J* = 1, Δ*F*_1_ = 0 and Δ*F* = 0 selection rules, the small splittings were disregarded and a single transition frequency value corresponding to the line center was considered. The assigned transition frequencies were least-squares fitted using the PGOPHER program^36^ employing an effective Hamiltonian for a linear molecule in a ^2^Π electronic state to obtain accurate ground state spectroscopic constants. The Hamiltonian contains rotational (*B*_0_), centrifugal distortion (*D*_0_), spin–orbit (*A*_0_ and *A*_*D*_0__), *Λ*-doubling (*p*_0_ and *q*_0_), magnetic hyperfine coupling due to the H and N nuclei (*a* and *b*_F_ for both, and *d* for N), as well as ^14^N nuclear electric quadrupole (*eQq*_0_ and *eQq*_2_) interaction terms. The result of the fit is summarized in Table 1. During the fitting procedure, most of the constants were determined, but those that could not be derived from the rotational data only had their values fixed to those previously obtained from the rovibrational measurements in paper I (Schmid *et al.*^15^), *i.e.*, *A*_0_, *A*_*D*_0__, and *p*_0_, which are also listed in Table 1 for comparison. The overall root-mean-square (rms) of the fit is 139 kHz, which is very reasonable for an open-shell molecule with coupled fine and hyperfine interactions. A line list containing the transition frequencies and fit residuals is given in the SI. A global fit of the rovibrational data reported in paper I^15^ of this series together with the rotational data of this work can be also found in the SI file for reference.

**Table 1:** Spectroscopic parameters of HCN^+^. The uncertainty in the last digits of each parameter is reported in parentheses. The values are given in MHz, unless otherwise noted

Parameter	IR^a^	Rot., this work^b^	Calc.^c^
*B*	40 554.6(3)	40 554.9424(68)	40 389.90
*D*	0.100(3)	0.09853(11)	0.084
*p*	731.6(18)	[731.6]^d^	
*q*	−60.0(2)	−60.039(11)	
*A*/cm^−1^	−49.3113(3)	[−49.3113]	
*A*_D_	−43.1(3)	[−43.1]	
*a*(*H*)		39.63(80)	26.07
*b*_F_(*H*)		−50.1(37)	−41.07
*a*(*N*)		38.75(36)	66.48
*b*_F_(*N*)		36.13(47)	32.20
*d*(*N*)		57.4(42)	80.18
*eQq*_0_(*N*)		−5.59(12)	−6.39
*eQq*_2_(*N*)		−16.26(67)	−8.81
*N*	137	71	
Max. *J*′	11.5	5.5	

a Ground state values from the paper I of this series, Schmid *et al.*^15^

b Ground state values determined in this work.

c Equilibrium values obtained at the CCSD(T)/aug-cc-pV5Z level of theory.

d Values in brackets, [], were kept fixed to those determined by Schmid *et al.*^15^

The values of the ground state constants determined in this work (Table 1) show excellent agreement with those from the rovibrational measurements of Schmid *et al.*^15^ The accuracy in the values of the *B*_0_, *D*_0_, and *q*_0_ constants was significantly refined, improving by two orders of magnitude. The hyperfine parameters for both the ^1^H and ^14^N nuclei are also determined and their values show reasonable agreement with their calculated counterparts, lending further support to our spectroscopic assignments.

## 4. Conclusions and outlook

The ground state rotational spectrum of HCN^+^ has been successfully measured and assigned for the first time. Together with the infrared spectroscopic data reported in the first paper of this series,^15^ the accurate ground state spectroscopic parameters and transition frequencies determined in this work (Table 1) will unquestionably allow searches of HCN^+^ in space not only *via* infrared astronomy, as recently shown for CH_3_^+^,^38^ but also through highly sensitive radio astronomy surveys targeted at the pure rotational spectra. A future astronomical detection of HCN^+^ would certainly contribute to enhance our current knowledge of the abundance and astrochemistry of interstellar CN-containing molecules.

Considering that neutral HCN and HNC are ubiquitous in various regions of the interstellar medium, including star-forming regions, photodissociation regions, diffuse clouds, translucent molecular clouds and starless cores,^8^ any of these regions could, in principle, serve as suitable candidates for searching for HCN^+^. In particular, the highly sensitive GOTHAM^39^ and QUIJOTE^40^ radio surveys have been showing that the cold Taurus Molecular Cloud, TMC-1, has an incredibly rich chemistry harboring many CN-containing molecules. Among those, the presence of neutral cyanopolyynes, HC_*n*_N, and of the recently detected HC_3_N^+^,^34^ HC_5_N^+^, and HC_7_N^+^ ^35^ cations in this source makes it a highly promising region for the search for HCN^+^. Unfortunately, no lines of HCN^+^ are currently covered by the GOTHAM and QUIJOTE line surveys. However, regardless of the candidate astronomical source, it is important to note that HCN^+^ may react quickly with H_2_ under astrophysical conditions, potentially affecting its overall abundance. Based on recent laboratory measurements,^9^ the rate coefficients determined for the reaction of both HCN^+^ and HNC^+^ isomers with H_2_ are close to the Langevin rate (≈10^−9^ cm^3^ s^−1^), and are also very similar to the rate derived for the CN^+^ + H_2_ reaction.^9^

Following the successful detection of the pure rotational fingerprints of HCN^+^ in this work, measurements of the rotational spectra of the lower-energy HNC^+^ isomer have also been of interest. The rotationally resolved infrared spectrum of the N–H stretching band of HNC^+^ (^2^Σ ← ^2^Σ) only became known after the measurements of this work had been carried out. This rovibrational band is described thoroughly in the first paper of this series and the accurate spectroscopic parameters obtained from the infrared measurements allowed us to observe very recently the ground state rotational spectrum of HNC^+^ for the first time, which will be detailed in a future publication. Apart from the high-resolution data reported here, novel spectroscopic investigations of HCN^+^ in the laboratory would also help to improve further the current spectroscopic models. This includes, for example, experiments at higher temperatures aiming to observe pure rotational transitions within the higher-energy ^2^Π_1/2_ spin–orbit ladder. Given that the impressive discoveries of the HC_3_N^+^,^34^ HC_5_N^+^ and HC_7_N^+^ ^35^ cations are solely based on predictions from quantum chemical calculations, laboratory experiments to refine the spectroscopic models are desirable.

## Conflicts of interest

There are no conflicts to declare.

## Supplementary Material

CP-028-D5CP04204F-s001

## Data Availability

All data supporting the findings of this study are available within the manuscript and its supplementary information (SI). Supplementary information is available. See DOI: https://doi.org/10.1039/d5cp04204f.

## References

[cit1] McGuire B. A. (2022). Astrophys. J., Suppl. Ser..

[cit2] Jefferts K. B., Penzias A. A., Wilson R. W. (1970). Astrophys. J., Lett..

[cit3] Gottlieb C. A., Brünken S., McCarthy M. C., Thaddeus P. (2007). J. Chem. Phys..

[cit4] Thorwirth S., Schreier P., Salomon T., Schlemmer S., Asvany O. (2019). Astrophys. J., Lett..

[cit5] Dixon T. A., Woods R. C. (1977). J. Chem. Phys..

[cit6] Agúndez M., Cernicharo J., Guélin M., Kahane C., Roueff E., Kłos J., Aoiz F. J., Lique F., Marcelino N., Goicoechea J. R., González Garca M., Gottlieb C. A., McCarthy M. C., Thaddeus P. (2010). Astron. Astrophys..

[cit7] Müller H. S. P., Thorwirth S., Roth D. A., Winnewisser G. (2001). Astron. Astrophys..

[cit8] Loison J.-C., Wakelam V., Hickson K. M. (2014). Mon. Not. R. Astron. Soc..

[cit9] Dohnal P., Jusko P., Jiménez-Redondo M., Caselli P. (2023). J. Chem. Phys..

[cit10] Ziurys L. M., Turner B. E. (1986). Astrophys. J..

[cit11] Ziurys L. M., Apponi A. J., Yoder J. T. (1992). Astrophys. J., Lett..

[cit12] Bogey M., Demuynck C., Destombes J. L. (1985). J. Chem. Phys..

[cit13] Amano T., Hashimoto K., Hirao T. (2006). J. Mol. Struct..

[cit14] Silva W. G. D. P., Bonah L., Schmid P. C., Schlemmer S., Asvany O. (2024). J. Chem. Phys..

[cit15] P. C. Schmid, S. J. P. Marlton, W. G. D. P. Silva, T. Salomon, J. Sarka, S. Thorwirth, O. Asvany and S. Schlemmer, Phys. Chem. Chem. Phys., 2025 10.1039/D5CP04201A PMC12851410 41604286

[cit16] Asvany O., Thorwirth S., Schmid P. C., Salomon T., Schlemmer S. (2023). Phys. Chem. Chem. Phys..

[cit17] Schmid P. C., Asvany O., Salomon T., Thorwirth S., Schlemmer S. (2022). J. Phys. Chem. A.

[cit18] Asvany O., Brünken S., Kluge L., Schlemmer S. (2014). Appl. Phys. B.

[cit19] Asvany O., Bielau F., Moratschke D., Krause J., Schlemmer S. (2010). Rev. Sci. Instr..

[cit20] Bast M., Böing J., Salomon T., Thorwirth S., Asvany O., Schäfer M., Schlemmer S. (2023). J. Mol. Spectrosc..

[cit21] Gupta D., Silva W. G. D. P., Doménech J. L., Plaar E., Thorwirth S., Schlemmer S., Asvany O. (2023). Faraday Discuss..

[cit22] Silva W. G. D. P., Cernicharo J., Schlemmer S., Marcelino N., Loison J. C., Agúndez M., Gupta D., Wakelam V., Thorwirth S., Cabezas C., Tercero B., Doménech J. L., Fuentetaja R., Kim W. J., de Vicente P., Asvany O. (2023). Astron. Astrophys..

[cit23] Silva W. G. D. P., Gupta D., Plaar E., Doménech J. L., Schlemmer S., Asvany O. (2023). Mol. Phys..

[cit24] Baddeliyanage C., Karner J., Melath S. P., Silva W. G. D. P., Schlemmer S., Asvany O. (2025). J. Mol. Spectrosc..

[cit25] Stanton J. F. , Gauss J., Cheng L., Harding M. E., Matthews D. A. and Szalay P. G., CFOUR, Coupled-Cluster techniques for Computational Chemistry, a quantum-chemical program package, With contributions from A.A. Auer, R.J. Bartlett, U. Benedikt, C. Berger, D.E. Bernholdt, Y.J. Bomble, O. Christiansen, F. Engel, R. Faber, M. Heckert, O. Heun, M. Hilgenberg, C. Huber, T.-C. Jagau, D. Jonsson, J. Jusélius, T. Kirsch, K. Klein, W.J. Lauderdale, F. Lipparini, T. Metzroth, L.A. Mück, D.P. O'Neill, D.R. Price, E. Prochnow, C. Puzzarini, K. Ruud, F. Schiffmann, W. Schwalbach, C. Simmons, S. Stopkowicz, A. Tajti, J. Vázquez, F. Wang, J.D. Watts and the integral packages MOLECULE (J. Almlöf and P.R. Taylor), PROPS (P.R. Taylor), ABACUS (T. Helgaker, H.J. Aa. Jensen, P. Jørgensen, and J. Olsen), and ECP routines by A. V. Mitin and C. van Wüllen. For the current version, see https://www.cfour.de

[cit26] Peterson K. A., Mayrhofer R. C., Woods R. C. (1990). J. Chem. Phys..

[cit27] Tarroni R., Mitrushenkov A., Palmieri P., Carter S. (2001). J. Chem. Phys..

[cit28] Dunning J., Thom H. (1989). J. Chem. Phys..

[cit29] Kendall R. A., Dunning J., Thom H., Harrison R. J. (1992). J. Chem. Phys..

[cit30] Gauss J., Lauderdale W. J., Stanton J. F., Watts J. D., Bartlett R. J. (1991). Chem. Phys. Lett..

[cit31] Watts J. D., Gauss J., Bartlett R. J. (1993). J. Chem. Phys..

[cit32] Frosch R. A., Foley H. M. (1952). Phys. Rev..

[cit33] Umeki H., Nakajima M., Endo Y. (2014). J. Chem. Phys..

[cit34] Cabezas C., Agúndez M., Endo Y., Tercero B., Marcelino N., de Vicente P., Cernicharo J. (2024). Astron. Astrophys..

[cit35] Cernicharo J., Cabezas C., Agúndez M., Endo Y., Tercero B., Marcelino N., de Vicente P. (2024). Astron. Astrophys..

[cit36] Western C. M. (2017). J. Quant. Spectrosc. Radiat. Transfer.

[cit37] Asvany O., Schlemmer S. (2009). Int. J. Mass Spectrom..

[cit38] Berné O., Martin-Drumel M.-A., Schroetter I., Goicoechea J. R., Jacovella U., Gans B., Dartois E., Coudert L. H., Bergin E., Alarcon F., Cami J., Roueff E., Black J. H., Asvany O., Habart E., Peeters E., Canin A., Trahin B., Joblin C., Schlemmer S., Thorwirth S., Cernicharo J., Gerin M., Tielens A., Zannese M., Abergel A., Bernard-Salas J., Boersma C., Bron E., Chown R., Cuadrado S., Dicken D., Elyajouri M., Fuente A., Gordon K. D., Issa L., Kannavou O., Khan B., Lacinbala O., Languignon D., Le Gal R., Maragkoudakis A., Meshaka R., Okada Y., Onaka T., Pasquini S., Pound M. W., Robberto M., Röllig M., Schefter B., Schirmer T., Sidhu A., Tabone B., Van De Putte D., Vicente S., Wolfire M. G. (2023). Nature.

[cit39] McGuire B. A., Burkhardt A. M., Loomis R. A., Shingledecker C. N., Kelvin Lee K. L., Charnley S. B., Cordiner M. A., Herbst E., Kalenskii S., Momjian E., Willis E. R., Xue C., Remijan A. J., McCarthy M. C. (2020). Astrophys. J., Lett..

[cit40] Cernicharo J., Agúndez M., Kaiser R. I., Cabezas C., Tercero B., Marcelino N., Pardo J. R., de Vicente P. (2021). Astron. Astrophys..

