# Supplementary material for: High-resolution spectroscopy of [H,C,N]+: II. Ground state rotational spectrum of HCN+ (X̃2Π)
Source: Phys Chem Chem Phys. 2025 Dec 3;28(7):4405–11. doi: 10.1039/d5cp04204f (PMC12757716; doi:10.1039/d5cp04204f)
Supplement: CP-028-D5CP04204F-s001 [file CP-028-D5CP04204F-s001.pdf]

Supplementary Information (SI) for Physical Chemistry Chemical Physics.
This journal is © the Owner Societies 2025

Electronic Supporting Information: High-resolution spectroscopy of $[H,C,N]^+$: II. Ground state rotational spectrum of $HCN^+(\tilde{X}^2\Pi)$

Weslley G. D. P. Silva,* Philipp C. Schmid, Divita Gupta, Sven Thorwirth, János Sarka, Oskar Asvany, and Stephan Schlemmer*

I. Physikalisches Institut, Universität zu Köln, Zülpicher Str. 77, 50937 Köln, Germany.

E-mail: silvaw@ph1.uni-koeln.de; schlemmer@ph1.uni-koeln.de

Overview of rotational measurements

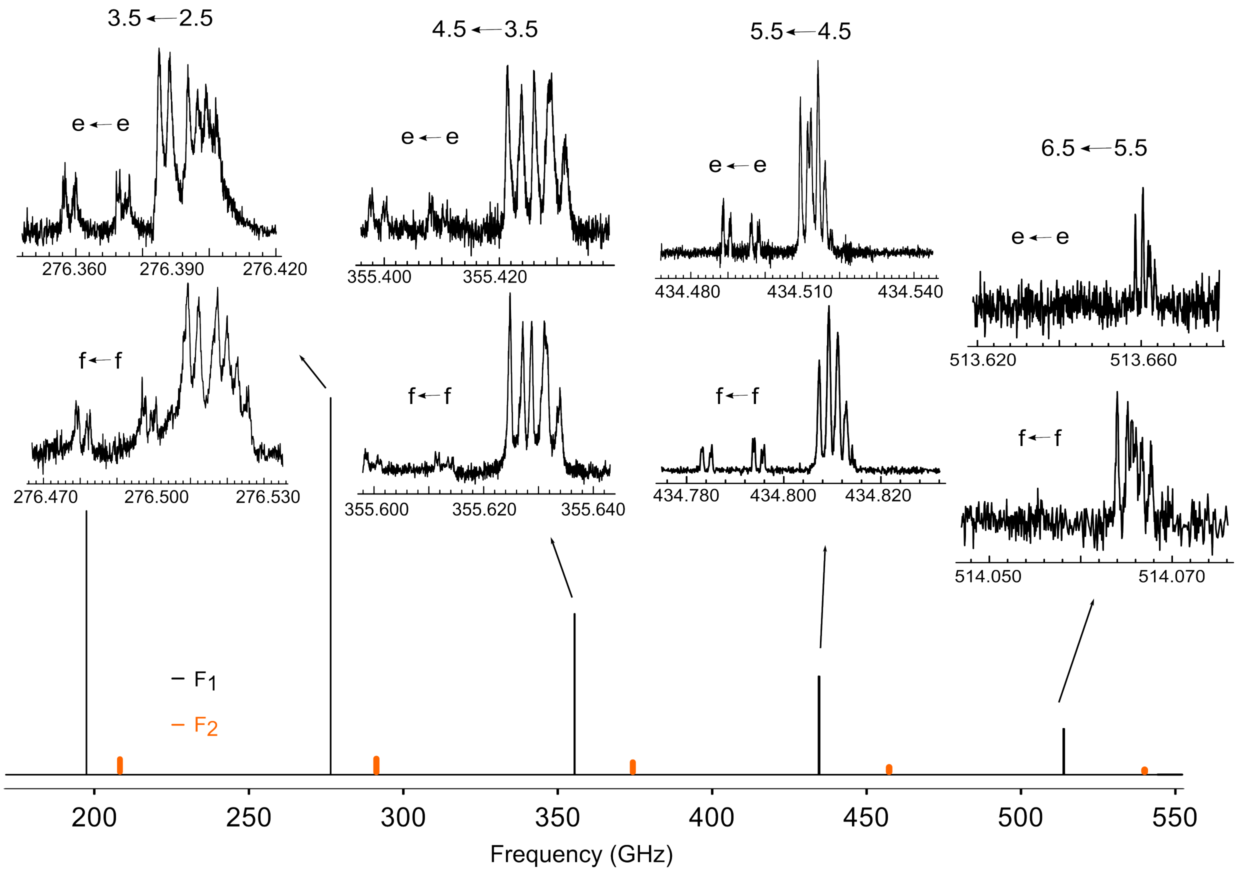

Figure S1: Overview spectrum of all rotational transitions measured in the 180-550 GHz frequency range. The rich hyperfine structure of HCN^+ is shown in detail for each Λ-doubling component ($e \leftarrow e$ and $f \leftarrow f$) in the insets.

Hyperfine structure assignments

As mentioned in the "Rotational Measurements" section of the main article, the pure rotational transitions of $HCN^+$are heavily affected by hyperfine and Zeeman effects. To exemplify the challenges caused by these effects on the spectral assignments further, we show once again in Figure S2 the $J' \leftarrow J'' = 3.5\ e \leftarrow 2.5\ e$ transition in detail. For each transition, the most intense peaks are those at higher frequencies which appear as six strong lines in the example of Figure S2. It is worth noting that the hyperfine splittings get smaller for transitions with higher J quantum numbers and the splitting becomes not fully resolved for some higher frequency transitions (see Figure S1). Although these strong features are broadened by the Zeeman effect (see orange trace below for the $3.5\ e \leftarrow 2.5\ e$ transition), their identities can still be recognized and the transition frequency of each broad peak can be unequivocally assigned to a distinct predicted hyperfine component (purple trace).

For the less intense transitions appearing on the left-side of the main peaks below 276,380 MHz (zoomed-in region of Figure S2), the assignments are more challenging. Our model predicts a total of eight hyperfine components in this range (see purple trace) and experimentally, there seems to be also roughly eight peaks, which resemble a quartet of doublets. However, four of these eight components are predicted to be extremely weak and not expected to appear in our low-temperature spectra. Thus, for the spectroscopic fits, we assumed that the experimental patterns seen for the overall less intense transitions, between 276,350 MHz and 276,380 MHz in this example, are Zeeman splittings of the four more intense components in this range. This seems to match quite well with the predictions accounting for the Zeeman effect (orange trace in Figure S2) and also with the transition intensities observed. Therefore, four transition frequencies corresponding to the center of each "doublet" were assigned to the four more intense components. For example, for the transitions labeled in the left-side of Figure S2, only the $F1', F' - F1'', F'' = 3.5, 4 - 3.5, 4$ and $3.5, 3 - 3.5, 3$ were included in the fit, while the $3.5, 3 - 3.5, 4$ and $3.5, 4 - 3.5, 3$ were neglected as they are expected to be extremely weak.

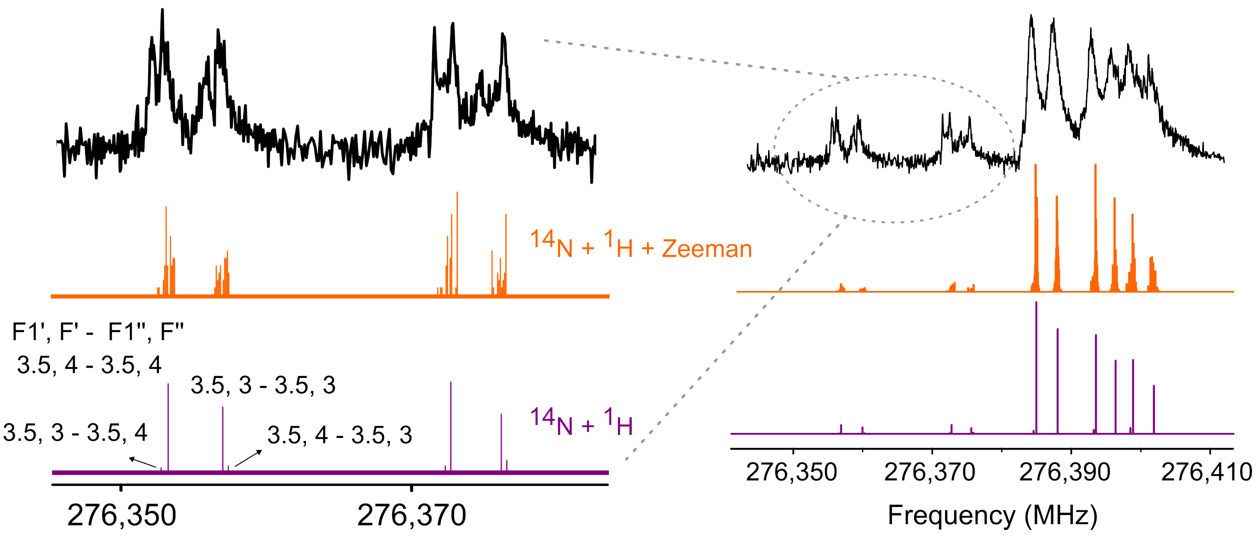

Figure S2: Pure rotational spectrum of the $J' \leftarrow J'' = 3.5\ e \leftarrow 2.5\ e$ transition of HCN^+. The experimental spectrum (in black) is compared to hyperfine model predictions including (in orange) and excluding (in purple) the Zeeman effect. The inset shows in detail the less intense hyperfine components in the $276,350 - 276.380$ MHz frequency range to highlight the influence of the external magnetic field on the observed spectra.

Transition frequencies and fit residuals

The line positions and fit residuals from the spectroscopic fit performed using the PGOPHER program. Hyperfine components which were not resolved in the spectra have been assigned to a single transition frequency value and fitted taking into account weighted averages. These lines can be found in the list right above the "Blend" label. Lines marked with *** were not included in the fit.

Observed	Obs-Calc	J'	N'	F1'	F2'	p		J"	N"	F1"	F2"	p
276356.6750	-0.0250	3.5	3	3.5	4	e	-	2.5	2	3.5	4	e
276359.8460	0.0395	3.5	3	3.5	3	e	-	2.5	2	3.5	3	e
276372.8850	0.0954	3.5	3	2.5	3	e	-	2.5	2	2.5	3	e
276375.5190	-0.0510	3.5	3	2.5	2	e	-	2.5	2	2.5	2	e
276385.1360	0.0984	3.5	3	4.5	5	e	-	2.5	2	3.5	4	e
276388.1850	0.0659	3.5	3	4.5	4	e	-	2.5	2	3.5	3	e
276393.8510	0.1717	3.5	3	3.5	4	e	-	2.5	2	2.5	3	e

276396.6520	0.1725	3.5	3	3.5	3	e	-	2.5	2	2.5	2	e
276399.1070	0.0596	3.5	3	2.5	3	e	-	2.5	2	1.5	2	e
276402.0390	0.0005	3.5	3	2.5	2	e	-	2.5	2	1.5	1	e
276479.0730	-0.0329	3.5	3	3.5	4	f	-	2.5	2	3.5	4	f
276482.1600	-0.0392	3.5	3	3.5	3	f	-	2.5	2	3.5	3	f
276497.1260	-0.0724	3.5	3	2.5	3	f	-	2.5	2	2.5	3	f
276500.1200	0.1591	3.5	3	2.5	2	f	-	2.5	2	2.5	2	f
276509.0880	-0.0412	3.5	3	4.5	5	f	-	2.5	2	3.5	4	f
276512.1370	-0.0588	3.5	3	4.5	4	f	-	2.5	2	3.5	3	f
276517.0310	-0.1592	3.5	3	3.5	4	f	-	2.5	2	2.5	3	f
276519.9060	-0.0675	3.5	3	3.5	3	f	-	2.5	2	2.5	2	f
276522.5310	-0.1045	3.5	3	2.5	3	f	-	2.5	2	1.5	2	f
276525.1690	***	3.5	3	2.5	2	f	-	2.5	2	1.5	1	f
355397.7580	-0.1758	4.5	4	4.5	5	e	-	3.5	3	4.5	5	e
355400.1430	-0.0992	4.5	4	4.5	4	e	-	3.5	3	4.5	4	e
355408.1600	-0.1393	4.5	4	3.5	4	e	-	3.5	3	3.5	4	e
355410.3870	-0.2247	4.5	4	3.5	3	e	-	3.5	3	3.5	3	e
355421.4707	-0.2052	4.5	4	5.5	6	e	-	3.5	3	4.5	5	e
355423.8234	-0.1958	4.5	4	5.5	5	e	-	3.5	3	4.5	4	e
355426.1126	-0.1588	4.5	4	4.5	5	e	-	3.5	3	3.5	4	e
355428.7777	0.2228	4.5	4	4.5	4	e	-	3.5	3	3.5	3	e
355428.7777	-0.4114	4.5	4	3.5	4	e	-	3.5	3	2.5	3	e
Blend	-0.0970											
355431.3580	-0.1633	4.5	4	3.5	3	e	-	3.5	3	2.5	2	e
355598.5700	0.0766	4.5	4	4.5	5	f	-	3.5	3	4.5	5	f
355600.8210	0.0365	4.5	4	4.5	4	f	-	3.5	3	4.5	4	f
355611.5010	0.0579	4.5	4	3.5	4	f	-	3.5	3	3.5	4	f

355613.8130	0.0773	4.5	4	3.5	3	f	-	3.5	3	3.5	3	f
355624.7784	0.2306	4.5	4	5.5	6	f	-	3.5	3	4.5	5	f
355627.0986	0.2263	4.5	4	5.5	5	f	-	3.5	3	4.5	4	f
355628.7059	0.1892	4.5	4	4.5	5	f	-	3.5	3	3.5	4	f
355631.2072	0.4261	4.5	4	4.5	4	f	-	3.5	3	3.5	3	f
355631.2072	-0.2276	4.5	4	3.5	4	f	-	3.5	3	2.5	3	f
Blend	0.0965											
355633.7380	-0.0103	4.5	4	3.5	3	f	-	3.5	3	2.5	2	f
434488.6930	-0.0006	5.5	5	5.5	6	e	-	4.5	4	5.5	6	e
434490.6260	0.0464	5.5	5	5.5	5	e	-	4.5	4	5.5	5	e
434496.2880	0.0676	5.5	5	4.5	5	e	-	4.5	4	4.5	5	e
434498.2840	0.1028	5.5	5	4.5	4	e	-	4.5	4	4.5	4	e
434509.4867	-0.1522	5.5	5	6.5	7	e	-	4.5	4	5.5	6	e
434511.5080	-0.0622	5.5	5	6.5	6	e	-	4.5	4	5.5	5	e
434512.2280	-0.2077	5.5	5	5.5	6	e	-	4.5	4	4.5	5	e
434514.2345	0.0420	5.5	5	4.5	5	e	-	4.5	4	3.5	4	e
434514.2345	-0.1220	5.5	5	5.5	5	e	-	4.5	4	4.5	4	e
Blend	-0.0394											
434516.0754	-0.0489	5.5	5	4.5	4	e	-	4.5	4	3.5	3	e
434783.1860	0.0630	5.5	5	5.5	6	f	-	4.5	4	5.5	6	f
434785.0550	0.0661	5.5	5	5.5	5	f	-	4.5	4	5.5	5	f
434793.8460	0.0539	5.5	5	4.5	5	f	-	4.5	4	4.5	5	f
434795.6658	-0.0654	5.5	5	4.5	4	f	-	4.5	4	4.5	4	f
434807.2777	0.2298	5.5	5	6.5	7	f	-	4.5	4	5.5	6	f
434809.2592	0.3017	5.5	5	6.5	6	f	-	4.5	4	5.5	5	f
434809.2592	0.0817	5.5	5	5.5	6	f	-	4.5	4	4.5	5	f
Blend	0.1928											

434811.0894	0.2238	5.5	5	4.5	5	f	-	4.5	4	3.5	4	f
434811.0894	0.0127	5.5	5	5.5	5	f	-	4.5	4	4.5	4	f
Blend	0.1190											
434812.7920	0.0154	5.5	5	4.5	4	f	-	4.5	4	3.5	3	f
513658.5904	0.0651	6.5	6	7.5	8	e	-	5.5	5	6.5	7	e
513660.4163	0.2256	6.5	6	7.5	7	e	-	5.5	5	6.5	6	e
513660.4163	0.0364	6.5	6	6.5	7	e	-	5.5	5	5.5	6	e
Blend	0.1316											
513663.3830	0.2154	6.5	6	5.5	5	e	-	5.5	5	4.5	4	e
513661.7300	0.2295	6.5	6	5.5	6	e	-	5.5	5	4.5	5	e
513662.1570	0.1097	6.5	6	6.5	6	e	-	5.5	5	5.5	5	e
514063.9980	-0.0723	6.5	6	7.5	8	f	-	5.5	5	6.5	7	f
514065.1060	-0.1183	6.5	6	6.5	7	f	-	5.5	5	5.5	6	f
514067.6760	-0.1825	6.5	6	5.5	5	f	-	5.5	5	4.5	4	f
514066.6890	-0.1792	6.5	6	6.5	6	f	-	5.5	5	5.5	5	f
514065.6000	-0.1122	6.5	6	7.5	7	f	-	5.5	5	6.5	6	f
514066.0230	-0.1910	6.5	6	5.5	6	f	-	5.5	5	4.5	5	f

Global fit

With the high-resolution rotational data of this work, we revisited the fit of the IR data of paper I (bands ν_1 and one component of the combination band $\nu_1 + \nu_2$), and performed a global fit. For simplicity, we fixed the B_0, D_0 and q_0 parameters of the ground state to our newly obtained values, and neglected the hyperfine splittings. The results can be found in Table S1 below. As can be seen, the spectroscopic parameters only change marginally. A more complete global fit, including also electronic transitions will be given in the paper III of this series (Marlton et al.).

Table S1: Spectroscopic parameters of HCN^+ for the ground state ($^2\Pi$ $(0,0,0)$), ν_1 state ($^2\Pi$ $(1,0,0)$), and $\nu_1 + \nu_2$ state μ component ($^2\Sigma^+$ $(1,1,0)$), obtained by a global fit of the spectra. All values are given in MHz, unless otherwise specified. Numbers in parentheses represent the uncertainty of the last digits.

Parameter [a]	paper I	This work [b]
B_0	40554.6(3)	40554.9424
D_0	0.100(3)	0.09853
p_0	731.6(18)	732.3(17)
q_0	-60.0(2)	-60.039
A_0 / cm^{-1}	-49.3113(3)	-49.3112(3)
A_{D0}	-43.1(3)	-43.4(2)
ν_1 / cm^{-1}	3056.3412(1)	3056.3412(1)
B_1	40273.7(3)	40274.1(1)
D_1	0.088(2)	0.088(1)
p_1	858.1(17)	858.6(17)
q_1	-70.6(2)	-70.6(1)
A_1 / cm^{-1}	-48.5987(3)	-48.5986(3)
A_{D1}	-40.9(2)	-41.0(2)
$\nu_1 + \nu_2$ / cm^{-1}	3340.8480(2)	3340.8480(2)
B_{1+2}	41069.6(4)	41069.7(3)
D_{1+2}	0.085(5)	0.081(4)
γ_{1+2}	-137.1(8)	-136.9(8)
rms / cm^{-1}	4.3×10^{-4}	4.4×10^{-4}

[a] For the two $^2\Pi$ states, p and q are the Λ doubling parameters, A the spin orbit constants, and A_D its distortion constants. For the $^2\Sigma$ state, γ represents the spin-rotation constant.
[b] The values of B_0, D_0 and q_0 were fixed to the values determined in Table 1 of the main paper.
